# Free access to medicines for the treatment of chronic diseases in Brazil

**DOI:** 10.1590/S1518-8787.2016050006118

**Published:** 2016-12-01

**Authors:** Noemia Urruth Leão Tavares, Vera Lucia Luiza, Maria Auxiliadora Oliveira, Karen Sarmento Costa, Sotero Serrate Mengue, Paulo Sergio Dourado Arrais, Luiz Roberto Ramos, Mareni Rocha Farias, Tatiane da Silva Dal Pizzol, Andréa Dâmaso Bertoldi

**Affiliations:** I Departamento de Farmácia. Faculdade de Ciências da Saúde. Universidade de Brasília. Brasília, DF, Brasil; II Departamento de Política de Medicamentos e Assistência Farmacêutica. Escola Nacional de Saúde Pública Sérgio Arouca. Fundação Oswaldo Cruz. Rio de Janeiro, RJ, Brasil; III Núcleo de Estudos de Políticas Públicas. Universidade Estadual de Campinas. Campinas, SP, Brasil; IV Programa de Pós-graduação em Epidemiologia. Universidade Federal do Rio Grande do Sul. Porto Alegre, RS, Brasil; V Departamento de Farmácia. Faculdade de Farmácia, Odontologia e Enfermagem. Universidade Federal do Ceará. Fortaleza, CE, Brasil; VI Departamento de Medicina Preventiva. Escola Paulista de Medicina. Universidade Federal de São Paulo. São Paulo, SP, Brasil; VII Departamento de Ciências Farmacêuticas, Centro de Ciências da Saúde. Universidade Federal de Santa Catarina. Florianópolis, SC, Brasil; VIII Departamento de Produção e Controle de Medicamentos. Faculdade de Farmácia. Universidade Federal do Rio Grande do Sul. Porto Alegre, RS, Brasil; IX Departamento de Medicina Social. Faculdade de Medicina. Universidade Federal de Pelotas. Pelotas, RS, Brasil

**Keywords:** Medicines, Equity in Access, Chronic Disease, National Drug Policy, Health Surveys

## Abstract

**OBJECTIVE:**

To analyze the free access to medicines for the treatment of chronic diseases in the Brazilian population, according to demographic and socioeconomic factors. We also analyzed the most used pharmacological groups, according to funding source: free-of-charge or out-of-pocket paid.

**METHODS:**

Analysis of data from the *Pesquisa Nacional sobre Acesso, Utilização e Promoção do Uso Racional de Medicamentos* (PNAUM – National Survey on Access, Use and Promotion of Rational Use of Medicines), a population-based household survey, of cross-sectional design, based on probabilistic sample of the Brazilian population. We analyzed as outcome the prevalence of free access (free-of-charge) to all medicines for treatment of the reported chronic diseases, in the last 30 days. We studied the following independent variables: sex, age group, education in complete years of school, economic class, health plan, and geographical region of residence. We estimated the prevalences and 95% confidence intervals (95%CI) and applied the Pearson’s Chi-squared test to assess the differences between the groups, considering a 5% significance level.

**RESULTS:**

About half of adults and older adults who have had full access to the treatment of chronic diseases in Brazil obtained all needed medicines for free (47.5%; 95%CI 45.1–50.0). The prevalences of free access were higher among men (51.4%; 95%CI 48.1–54.8), age group of 40-59 years (51.1%; 95%CI 48.1–54.2), and in the poorest social classes (53.9%; 95%CI 50.2–57.7). The majority of medicines that act on the cardiovascular system, such as diuretics (C03) (78.0%; 95%CI 75.2–80.5), beta-blockers (C07) (62.7%; 95%CI 59.4–65.8), and the agents that work in the renin-angiotensin system (C09) (73.4%; 95%CI 70.8–75.8), were obtained for free. Medicines that act on the respiratory system, such as agents against obstructive airway diseases (R03) (60.0%; 95%CI 52.7–66.9) were mostly paid with own resources.

**CONCLUSIONS:**

Free access to medicines for treatment of chronic diseases occurs to a considerable portion of the Brazilian population, especially for the poorest ones, indicating decreased socioeconomic inequalities, but with differences between regions and between some classes of medicines.

## RESUMO

**OBJETIVO:**

Analisar o acesso gratuito ao tratamento medicamentoso para doenças crônicas na população brasileira, segundo fatores socioeconômicos e demográficos. Analisaram-se também os grupos farmacológicos mais utilizados, segundo fonte de financiamento: gratuito ou pago do próprio bolso.

**MÉTODOS:**

Análise de dados oriundos da Pesquisa Nacional sobre Acesso, Utilização e Promoção do Uso Racional de Medicamentos (PNAUM), inquérito domiciliar de base populacional, de delineamento transversal, baseado em amostra probabilística da população brasileira. O desfecho analisado foi a prevalência de acesso gratuito (sem pagamento) a todos os medicamentos para tratamento das doenças crônicas referidas, nos últimos 30 dias. As variáveis independentes investigadas foram: sexo, faixa etária, escolaridade em anos completos de estudo, classe econômica, plano de saúde e região geográfica de residência. Foram estimadas as prevalências e calculados intervalos de 95% de confiança (IC95%) e aplicado o teste Qui-quadrado de Pearson para avaliação das diferenças entre os grupos, considerando nível de significância de 5%.

**RESULTADOS:**

Cerca de metade dos adultos e idosos que tiveram acesso total ao tratamento de doenças crônicas no Brasil obtiveram todos os medicamentos que necessitavam gratuitamente (47,5%; IC95% 45,1–50,0). As prevalências de acesso gratuito foram maiores entre os homens (51,4%; IC95% 48,1–54,8), na faixa etária de 40-59 anos (51,1%; IC95% 48,1–54,2) e nas classes sociais mais pobres (53,9%; IC95% 50,2–57,7). Grande parte dos medicamentos que atuam no sistema cardiovascular, como os diuréticos (C03) (78,0%; IC95% 75,2–80,5), betabloqueadores (C07) (62,7%; IC95% 59,4–65,8) e os agentes que atuam no sistema renina-angiotensina (C09) (73,4%; IC95% 70,8–75,8) foram obtidos de forma gratuita. Os medicamentos que atuam no sistema respiratório como os agentes contra doenças obstrutivas das vias aéreas (R03) (60,0%; IC95% 52,7–66,9) foram na sua maioria pagos do próprio bolso.

**CONCLUSÕES:**

O acesso gratuito aos medicamentos para tratamento das doenças crônicas ocorre para uma considerável parcela da população brasileira, principalmente para os mais pobres, indicando diminuição das desigualdades socioeconômicas, mas com diferenças regionais e entre algumas classes de medicamentos.

## INTRODUCTION

Access to medicines is a duty of the Brazilian State, which incorporated, among its principles and constitutional guidelines, the guarantee of comprehensive therapeutic care, including pharmaceutical services within the Brazilian Unified Health System (SUS). This access occurs by the availability of medicines in health-care networks, their geographical accessibility and acceptability, promoting the rational use of the products^3^.

Different strategies have been developed in Brazil to implement the guidelines of the National Drug Policy and National Pharmaceutical Services Policy. Among the initiatives, we highlight the structuring of pharmaceutical services, the advances of regulatory frameworks concerning access to medicines in SUS, the improvement of the organization of public pharmaceutical services funding, and the expansion of federal resources, among others^17^.

The public spending with medicines is growing and is the second largest item of the health-care systems total expenditure, surpassed only by hospital care^13^. Between 2003 and 2014, the budget of the Brazilian Ministry of Health for pharmaceutical services started from 1.9 billion to 12.4 billion Brazilian Reals, with an investment exceeding 80 billion in the area at the time^17^. Nevertheless, medicines also represent an important item of households health spending, particularly for low-income families and, despite the reduction of expenditure with medicines on their income observed in some studies, this is still the main component of health spending^6^ ^,^ ^9^.

Brazil lives a singular transition, manifested in the coexistence of infectious and parasitic diseases, reproductive health problems, external causes, and chronic diseases^13^, and this epidemiological profile requires growing medicine consumption by the population.

Chronic non-communicable diseases are considered one of the most challenging problems of world public health^24^. They are the main causes of death in the world, accounting for 68.0% of deaths in 2012. Approximately 75.0% of deaths from these diseases occur in low and middle-income countries, and 40.0% are considered premature deaths (before 70 years)^26^. In Brazil, chronic non-communicable diseases have become the top priority of public health policies^22^, since they affect, more intensely, the most vulnerable segments of the Country, with the lowest income and schooling^2^.

The access to medicines and the guarantee of adequate pharmacological treatment promote more effective control of chronic non-communicable diseases, which enables the reduction of morbidity and mortality and improves users’ health and quality of life^10^.

However, epidemiological studies regarding access to free medicines in the Country are still scarce. Moreover, national studies use different methods, diverse target population and local coverage, which makes difficult comparative analysis of their results and to evaluate the impacts of recent pharmaceutical public interventions in the Country^1^ ^,^ ^19^ ^,^ ^21^ ^,^ ^a^.

This study aimed to analyze the free access to pharmacological treatment for chronic diseases in the Brazilian population, according to demographic and socioeconomic factors. We also analyzed the most used pharmacological groups, according to source of funding: free or paid with own resources.

## METHODS

The data analyzed in this study came from the National Survey on Access, Use and Promotion of Rational Use of Medicines (PNAUM), a population-based household survey, of cross-sectional design, based on probabilistic sample of the Brazilian population. Data collection was carried out between September 2013 and February 2014. The population under study was of individuals of all ages living in private permanent homes in the urban area of the Brazilian territory. We conducted face-to-face interviews in households with application of questionnaires, and data were collected in electronic devices.

The instruments used were developed by a group of researchers from Brazilian universities, experts in the field, and standardized and tested before being implemented. The sampling process was complex and resulted in representative sample of Brazil and its five geographical regions, stratified by sex and age groups. Details about the sampling and logistics of data collection can be found in the methodological article of PNAUM^16^.

The analyses of this paper were carried out with two approaches using two databases with different denominators. One database refers to the sample of adult population aged 20 years or more who reported some chronic disease with more than six months duration in the period prior to the interview. The research on access to medicines included all interviewers who reported medical indication of medicines for the reported chronic non-communicable diseases (n = 12,725), from the question: “In the last 30 days, were you without some of these medicines for a while?” Those who reported not being without any of the medicines prescribed by the doctor were considered with full access to treatment (n = 10,782). For each reported medicine for treatment of chronic diseases, we asked if the product has been obtained for free or paid of out-of-pocket.

We analyzed the outcome of prevalence of free access (without pay) to all medicines for treatment of the reported chronic diseases, in the last 30 days.

We investigated the following independent variables: sex (female; male); age group (20-39; 40-59; 60 or more); education in complete years of schooling (0-8; 9-11; 12 or more); ABEP economic classification (http://www.abep.org/) (A/B; C; D/E); health plan (yes; no), and geographical region of residence (North; Northeast; Southeast; South; Midwest). We estimated the prevalences and 95% confidence intervals (95%CI) and applied the Pearson’s Chi-squared test to assess the statistical significance of differences between the groups, considering a 5% significance level.

The other database is composed of medicines reported by the population aged 20 years or more for the treatment of chronic diseases (n = 30,256). For the analysis, the medicines were stratified by funding source (free or paid of out-of-pocket), and classified according to levels 1 and 2 of the ATC (Anatomical Therapeutic Chemical [ATC] classification system)^25^, and by presence in the *Relação Nacional de Medicamentos Essenciais* (RENAME – National List of Essential Medicines) in force at the time of data collection.

All analyses were performed with the statistical package SPSS 18.0, using the set of CSPLAN commands suitable for the analysis of complex samples and ensuring the necessary weight to the sample size.

This study was submitted and approved by the National Commission of Ethics in Research (Opinion 398,131, September 16, 2013). All participants (or responsible ones, in the case of people unable to answer their own questionnaire) signed an informed consent form.

## RESULTS

**Figure 1:**
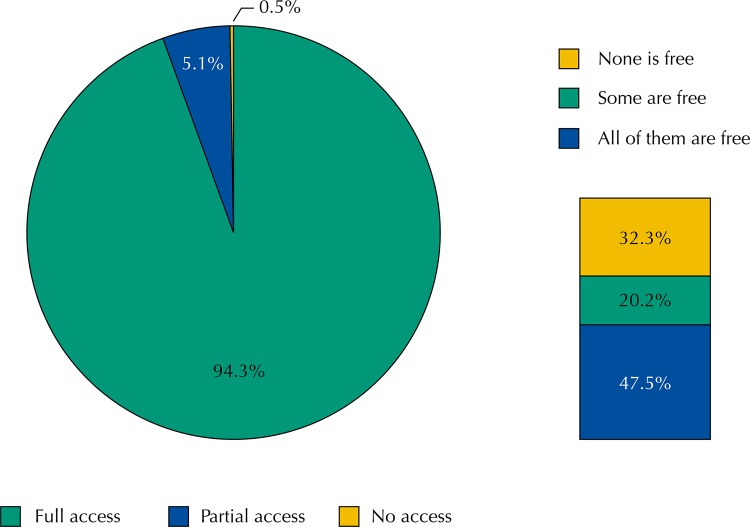
Prevalence of access to treatment of chronic diseases in Brazil and prevalence of free access, among those who reported full access to pharmacological treatment.* PNAUM, Brazil, 2014.

The majority of people aged 20 years or more who reported medical indication and use of medicines to treat chronic diseases (94.3%; 95%CI 93.4–95.1) obtained all the medicines they needed in the last 30 days; a small proportion of them (5.1%; 95%CI 4.4–6.0) obtained some of the medicines they needed, and only 0.5% (95%CI 0.4–0.7) failed to obtain any medicine for their treatment in the past month (Figure 1).

* Percentages weighted by sample weights and post-stratification according to age and sex.

Among those who got full access to treatment, about half of them got all medicines for free (47.5%; 95%CI 45.1–50.0), a small part got some free medicine (20.2%; 95%CI 19.0–21.4), and 1/3 (32.3%; 95%CI 30.1–34.6) paid of out-of-pocket for all medicines to treat chronic diseases (Figure 1).

Regarding socioeconomic and demographic characteristics, men and individuals aged 40-59 years showed higher prevalence of free access to all medicines. As for education, the prevalence of free access was similar among individuals (Table 1). Regarding economic class, the poorest (classes D/E) had greater access to all free medicines to treat the reported chronic diseases, as well as those who did not have health plan in relation to who had a plan or private insurance (Table 1).

**Table 1:** Prevalence of free access to all medicines for adults and older adults who have had full access to the treatment of chronic diseases, according to socioeconomic and demographic variables. PNAUM, Brazil, 2014.

Variable	Prevalence of free access to all medicines		

	%^a^	95%CI	P^b^
Demographic Characteristics			

Sex			< 0.001
Male	51.4	48.1–54.8	
Female	45.3	42.9–47.8	
Age (years)			0.001
20-39	41.2	35.9–46.8	
40-59	51.1	48.1–54.2	
≥ 60	46.0	43.0–49.0	

Socioeconomic characteristics			

Education (years of schooling)			0.933
0-8	47.5	44.6–50.6	
9-11	47.0	43.7–50.4	
≥ 12	47.9	43.6–52.2	
Economic classification (ABEP)^c^			< 0.001
A/B	35.8	32.6–39.2	
C	50.5	47.7–53.3	
D/E	53.9	50.2–57.7	
Region of the country			0.074
North	53.0	47.0–58.9	
Northeast	43.4	40.1–46.7	
Southeast	49.0	44.8–53.3	
South	47.8	44.0–51.6	
Midwest	44.8	41.1–48.5	
Has health plan			< 0.001
Yes	27.6	24.9–30.5	
No	55.7	53.1–58.1	

Total	47.5	45.1–50.0	

^a^ Percentages weighted by sample weights and post-stratification according to age and sex.

^b^ Pearson’s Chi-squared test.

^c^ Associação Brasileira das Empresas Brasileiras (ABEP). Critério de classificação econômica Brasil. São Paulo: ABEP; 2013. Available from: http://www.abep.org

Concerning geographical regions of the country, people living in the North and Southeast had higher prevalence of free access to all medicines; the Northeast region presented the lowest prevalence (Table 1).

**Figure 2:**
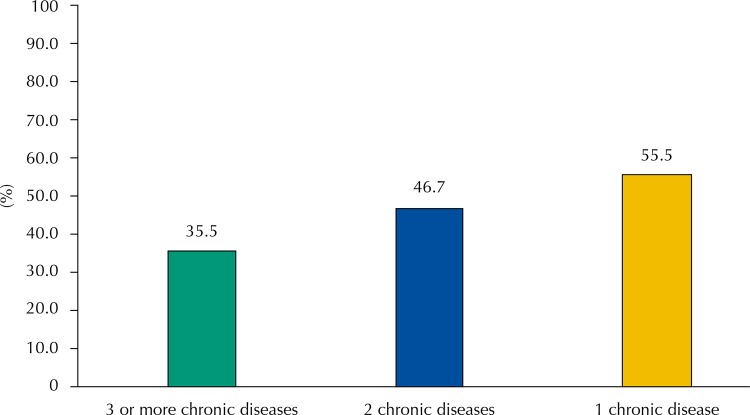
Prevalence of free access to all medicines according to the number of chronic diseases reported by Brazilians adults and older adults who reported full access to treatment for chronic diseases.* PNAUM, Brazil, 2014.

Among adults and older adults who reported full access to treatment, more than half reported more than one chronic disease at the time of the interview: 27.1% (95%CI 25.8–28.4) reported having two chronic diseases and 27.4% (95%CI 25.7–29.2), three or more (data not presented). Stratifying free access by the number of reported diseases: those with greater number of diseases have had lower prevalence of free access to all medicines (35.5%; 95%CI 32.5–38.6) than those who reported one disease (55.5%; 95%CI 52.4–58.6) (Figure 2).

* Percentages weighted by sample weights and post-stratification according to age and sex.

**Figure 3:**
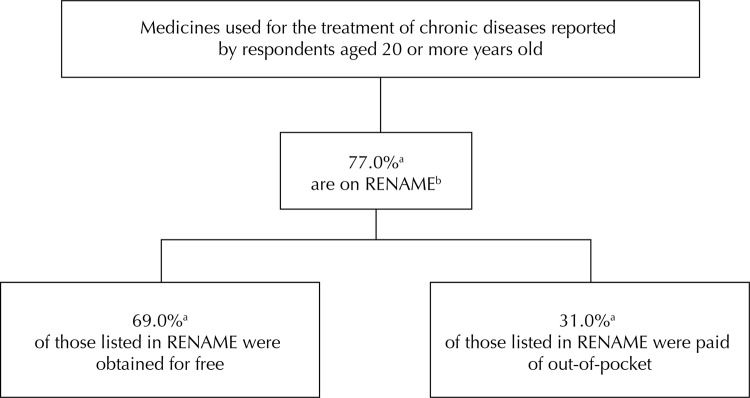
Classification of the medicines reported for treatment of chronic diseases of adults and older adults in Brazil according to RENAME^b^ and funding source (free or paid of out-of-pocket). PNAUM, Brazil, 2014.

^a^ Percentage adjusted by sample weights and post-stratification according to age and sex.

^b^ RENAME - Relação de Medicamentos Essenciais 2013, 8ª Edição, 2014. Available from: http://www.youblisher.com/p/941590-Rename/

Analyzing the pharmacological groups of medicines referred for treatment of chronic diseases, we verified that the vast majority is included in the existing RENAME, and, of these, a large part was obtained for free (69.0%; 95%CI 66.9–71.0) (Figure 3).

Stratifying the more frequent pharmacological groups for treatment of chronic diseases, most medicines that act on the cardiovascular system, such as diuretics (C03) (78.0%; 95%CI 75.2–80.5), beta-blockers (C07) (62.7%; 95%CI 59.4–65.8), and agents that work in the renin-angiotensin system (C09) (73.4%; 95%CI 70.8–75.8), were obtained for free. On the other hand, medicines that act on the respiratory system, such as agents against obstructive airway diseases (R03) (60.0%; 95%CI 52.7–66.9), were mostly paid of out-of-pocket (Table 2).

**Table 2:** Groups of the ATC^a^ classification more used by adults and older adults for the treatment of chronic diseases according to funding source of the medicines (free of charge or paid of out-of-pocket). PNAUM, Brazil, 2014.

ATC classification levels 1 and 2	Funding source of the medicine			

	% paid^b^	95%CI	% free^b^	95%CI
A: Digestive apparatus and metabolism				
A10 Medicine used for diabetes	21.4	18.7–24.4	78.6	75.6–81.3
C: Cardiovascular system				
C01 Cardiac therapy	56.2	49.5–62.6	43.8	37.4–50.5
C02 Antihypertensives	50.4	41.7–59.1	49.6	40.9–58.3
C03 Diuretics	22.0	19.5–24.8	78.0	75.2–80.5
C07 Beta-blockers	37.3	34.2–40.6	62.7	59.4–65.8
C0 Calcium channel blockers	41.4	36.3–46.7	58.6	53.3–63.7
C09 Agents acting on the renin-angiotensin system	26.6	24.2–29.2	73.4	70.8–75.8
C10 Lipids modifier agents	44.6	40.7–48.6	55.4	51.4–59.3
N: Nervous system				
N06 Psychoanaleptics	41.5	37.5–45.6	58.5	54.4–62.5
N03 Antiepileptics	41.2	36.0–46.7	58.8	53.3–64.0
N05 Psycholeptics	45.3	39.1–51.6	54.7	48.4–60.9
N04 Antiparkinson	26.4	17.2–38.2	73.6	61.8–82.8
R: Respiratory system				
R03 Agents against airway obstructive diseases	60.0	52.7–66.9	40.0	33.1–47.3
R06 Antihistamines for systemic use	56.6	43.3–69.1	43.4	30.9–56.7

ATC: Anatomical Therapeutic Chemical classification system

^a^ ATC levels 1 and 2.

^b^ Percentages adjusted by sample weights and post-stratification according to age and sex.

## DISCUSSION

This study identified that about half of adults and older adults who have had full access to the treatment of chronic diseases in Brazil got all medicines they needed for free. The prevalence of free access occurs differently between men and women and between Brazil’s regions, being higher in the North and Southeast regions and in poorer social classes.

This is the first population-based study that investigates the access to medicines with representative sample of the population living in the urban area of the five regions of Brazil, allowing the generalization of results for the Brazilian population. As methodological strategy, we analyzed the free access to the treatment of chronic diseases in those who reported full access to treatment, analyzing the socioeconomic and demographic differences in the Country, as well as the source of funding for pharmacological groups most used by the Brazilian population.

Considering obtaining from all sources of funding, the prevalence of full access to the treatment of chronic diseases was high, similar to that found in other studies carried out in Brazil, despite the limitation of comparisons due to differences of recall periods and study approaches^4^ ^,^ ^8^ ^,^ ^19^ ^,^ ^20^. The access level found matched the results of the World Health Survey, conducted in 2002 and 2003 in 70 countries. In this investigation, 72.0% to 85.0% of respondents reported obtaining all or most of the medicines for conditions treatable in the last 12 months^23^.

The prevalence found here confirms the results of the National Household Sample Survey (PNAD) conducted in 2008 in Brazil by the Brazilian Institute of Geography and Statistics (IBGE). PNAD assessed the population that had medicines prescribed in SUS, of which 45.3% obtained them in the public system^7^. We need caution when comparing these data, given that PNAD analyzed only the access of prescriptions from SUS, and this study examined all sources; however, both results indicate that about half of the Brazilian population gets free access to treatment since 2008.

Considering more prevalent chronic diseases, as hypertension and diabetes, recent publications point out the role of SUS as an important provider of medicines. The Surveillance System of Risk and Protection Factors for Chronic Diseases by Telephone Survey (VIGITEL) noted that, in the analyzed period (2011-2013), about 60.0% of medicines to control hypertension and more than 70.0% of diabetes medicines were obtained for free by adults (≥ 18 years), whether by the primary healthcare unit or by the *Farmácia Popular* (Popular Pharmacy Program)^18^. The analysis of the National Survey on Health (PNS 2013) showed that the vast majority of patients using medicines for hypertension has a single source for obtaining them, with about half of the supply of medicines by SUS (whether by public health system’s pharmacies or by the Popular Pharmacy Program), one third exclusively by private pharmacies, and small participation by private health plans^15^.

Free access to all medicines to treat chronic diseases reported in the last month differed according to socioeconomic and demographic characteristics. The poorest had greater free access to all medicines to treat chronic diseases, strengthening the hypothesis that SUS is promoting equity in access to medicines^19^. This finding is important, considering the inverse association between socioeconomic position and under-utilization of medicines, and that, in these population sectors, the public health system is often the only alternative to enable the needed therapy^6^ ^,^ ^7^ ^,^ ^14^.

Regarding the differences in the five regions of the Country, the Northeast region showed the lowest prevalence of free access to all medicines, which has been observed in prior studies^7^ ^,^ ^21^. Considering that, in the Northeast region, 78.0% of cities have low Human Development Index (HDI) of Income^b^ and that the income expenditure on purchasing medicines can be almost three times higher among the poorest (7.3%), when compared to the richest (2.7%)^6^, these results may indicate that the population of this region is more vulnerable to treatment discontinuation because of inability to pay for treatment with medicines for chronic diseases^11^.

Chronic non-communicable diseases are the main sources of disease burden in Brazil, and important policies to their prevention and control have been implemented, including the expansion of access to medicines policies, such as the free treatment for hypertension, diabetes, and asthma by the Popular Pharmacy Program^22^. In this study, we highlight the importance of these policies on access to medicines in the Country. However, free access to medicines in Brazil is lower for individuals with higher number of chronic diseases, indicating that full access to treatment is still a gap to be addressed by the managers of the health-care network.

To implement free access for the population, the deficiencies found in studies in recent years relating to the management of pharmaceutical services^5^ ^,^ ^11^ ^,^ ^14^, such as the low availability of medicines in public health units, must be focus of interventions of SUS managers, toward the promotion of free access to medicines in the Country. Moreover, we stress that, in this study, most medicines obtained for free by the Brazilian population were included in the existing RENAME, a guiding instrument for the selection and care of diseases and harms within the SUS, showing that the targeting of this use of medicines rationalization strategy complies with the needs of the population.

Cardiovascular diseases, despite their reduction, are the leading cause of death in Brazil^22^. In this study, most medicines used for the treatment of these diseases by the Brazilian population were obtained for free, a number higher than that of a study carried out previously in two Brazilian regions^21^. This result may suggest that the expansion of access strategies for pharmaceutical services reorganization and funding implemented in the Country in recent years have been effective in increasing free access to these medicines for the Brazilian population. However, the high out-of-pocket expenditure on medicines for respiratory system related conditions show gaps on access to this group of medicines, which are used for chronic diseases such as asthma and involve access to health services and diagnostics.

We can conclude that free access to medicines for treatment of chronic non-communicable diseases has been effective to a considerable portion of the Brazilian population, especially for the poorest ones, indicating decreased socioeconomic inequalities, but with differences between regions and between classes of medicines.

## Footnotes

a Costa KS. Acesso e uso de medicamentos: inquéritos de saúde como estratégia de avaliação [these]. Campinas (SP): Faculdade de Ciências Médicas da Universidade Estadual de Campinas; 2014.

b Programa Nacional das Nações Unidas para o desenvolvimento (PNUD). Atlas do desenvolvimento humano no Brasil 2013: IDHM Renda. Brasília (DF); 2013 [cited 2014 Sept 20]. Available from: http://www.pnud.org.br/arquivos/ idhm-renda.pdf
